# Ischemic heart disease mortality due to fine particulate matter in Seoul between 2016 and 2020

**DOI:** 10.1186/s12889-025-24204-y

**Published:** 2025-10-22

**Authors:** Jongmin Oh, Myung-Sook Park, Yun-Chul Hong, AiMS-CREATE, Ho Kim, Whanhee Lee, Ejin Kim, Cinoo Kang, Insung Song, Hyemin Jang, Jieun Min, Dohoon Kwon, Jieun Oh, Jeongmin Moon, Jinah Park, Ayoung Kim, Seoyeong Ahn

**Affiliations:** 1 Department of Environmental Medicine, College of Medicine, Ewha Womans University, Seoul, Republic of Korea; 2 Institute of Ewha-SCL for Environmental Health (IESEH), College of Medicine, Ewha Womans University, Seoul, Republic of Korea; 3 Department of Human Systems Medicine, College of Medicine, Seoul National University, Seoul, Republic of Korea; 4 Department of Preventive Medicine, College of Medicine, Seoul National University, Seoul, Republic of Korea; 5 Institute of Environmental Medicine, Seoul National University Medical Research Center, 103 Daehakro, Jongno-Gu, Seoul, 110-799 Republic of Korea; 6 Seoul Metropolitan Government Environmental Health Center, Seoul, Republic of Korea

**Keywords:** Mortality burden, Health impact assessment, Health benefit, Fine particulate matter, Korea

## Abstract

**Background:**

Ischemic heart disease (IHD) continues to rank among the leading global causes of mortality, consistently linked to long-term exposure to fine particulate matter (PM_2.5_). Despite a declining trend in the annual average PM_2.5_ concentration in Seoul, they still fail to meet the air quality standards set by the Korean Ministry of Environment (15 µg/m^3^). We aim to estimate the IHD mortality attributable to PM_2.5_ and the health benefits derived from reducing PM_2.5_ concentration in Seoul, South Korea, between 2016 and 2020.

**Methods:**

We utilized machine-learning-based PM_2.5_ data, Global Exposure Mortality Model, mortality data, and population data to estimate the burden of IHD mortality attributed to PM_2.5_ and the benefits in IHD mortality associated with reductions in PM_2.5_ concentration in Seoul between 2016 and 2020. We also estimated the health benefits by considering the reference concentration in three reduction scenarios.

**Results:**

During the study period, the average PM_2.5_ concentration was 23.5 µg/m^3^, with 10,971 IHD deaths among individuals aged ≥ 25 years. We estimated 2,861 excess IHD deaths (aged ≥ 25 years) attributable to PM_2.5_ exposure between 2016 and 2020. Under the PM_2.5_ reduction scenario, achieving a PM_2.5_ concentration of 15 µg/m^3^ corresponds to an avoidable mortality rate of approximately 8%.

**Conclusions:**

Our study assessed the burden of IHD mortality attributable to ambient PM_2.5_ and the potential health benefits associated with its reduction in Seoul between 2016 and 2020. The findings suggest that continued efforts to reduce PM_2.5_ concentrations could significantly mitigate IHD mortality, particularly in the context of an aging population.

**Supplementary Information:**

The online version contains supplementary material available at 10.1186/s12889-025-24204-y.

## Introduction

Ischemic heart disease (IHD) is the leading cause of death and a major global public health concern [1–4]. According to the Global Burden of Disease (GBD) study, the total number of disability-adjusted life years (DALYs) owing to IHD has steadily increased since 1990, reaching an estimated 182 million DALYs and 9.14 million deaths in 2019 [3].

Cardiovascular disease (CVD) is the leading cause of death in most regions globally. However, in parts of East Asia, including Korea, it is the second leading cause of death after neoplasm [4]. In 2020, the mortality rate from CVD in Korea was 63 deaths per 100,000 population. CVD mortality, including IHD, decreased between 1983 and 2008 but has been increasing since then [5]. However, age-standardized CVD mortality rates have been decreasing annually. Lee et al. suggested the that the reason for this change could be attributed to population aging [5].

Fine particulate matter (PM_2.5_) is particulate matter with a diameter of less than 2.5 micrometers. Exposure to PM_2.5_ has been recognized as a risk factor for mortality and cardiovascular diseases [6–8]. Specifically, long-term exposure to PM_2.5_ is a recognized risk factor for IHD events and mortality [6–12]. Furthermore, Korean studies have clarified the link between long-term exposure to PM_2.5_ and ischemic mortality [13, 14].

Seoul, covering an area of approximately 605 km^2^ and home to a population of 10 million residents, is one of the most densely populated metropolitan cities in South Korea. In 2020, the Korean Statistical Office reported 14,114 deaths from IHD in Korea. Among these, Seoul accounted for 18% of the total IHD deaths, indicating a significant mortality burden.

The PM_2.5_ concentration in Seoul decreases annually; however, it still fails to meet the air quality standards (15 µg/m^3^) set by the Korean Ministry of Environment (KMOE) [15]. Furthermore, the World Health Organization (WHO) updated its air quality guideline (AQG) in 2021 [16], recommending that the annual PM_2.5_ concentration should not exceed at 5 µg/m^3^. Understanding the burden of IHD mortality due to exposure to ambient PM_2.5_ is crucial, warranting proactive measures to meet international air quality standards.

Although the GBD studies and Institute for Health Metrics and Evaluation have provided valuable global estimates of disease burden attributable to ambient PM_2.5_, these estimates are largely based on modeled data for population, exposure levels, and mortality. Consequently, the attributable mortality burden may be either under- or overestimated. To enhance the precision of burden estimates, the use of high-resolution data on population, exposure, and health outcomes at the local level is essential.

In this context, the present study estimates IHD mortality attributable to PM_2.5_ in Seoul using observed mortality data and high-resolution exposure and population data. This approach enhances the reliability of the findings and contributes to more accurate, location-specific evidence for public health and environmental policy.

Therefore, this study aimed to calculate the IHD mortality attributable to PM_2.5_ in Seoul, South Korea, between 2016 and 2020. Furthermore, we estimated the health benefits associated with reductions in PM_2.5_ for IHD mortality.

## Materials and methods

We estimated the health impact assessment of PM_2.5_ concentration in 25 administrative districts (known as gu) in Seoul, South Korea, between 2016 and 2020. This involved utilizing mortality data, population data, PM_2.5_ exposure, and exposure-response function.

### Data collection

#### Mortality

We utilized Causes of Death Statistics from Microdata Integrated Service (https://mdis.kostat.go.kr/index.do) between 2016 and 2020. This data enables us to define IHD deaths according to the International Classification of Diseases-10 (ICD-10; I20–I25). Of the 221,042 deaths in Seoul, 10,972 cases of IHD deaths were identified. Among these, only one case of death occurred under the age of 25 years. To evaluate the health impact assessment of ambient PM_2.5_, we extracted individuals aged ≥ 25 years from the IHD deaths (*n* = 10,971). We categorized the number of IHD deaths using year and age group (25–29, 30–34, 35–39, …, and > 80).

#### Population data

We gathered population data from the Korean Statistical Information Service (https://kosis.kr/statHtml/statHtml.do?orgId=101&tblId=DT_1B040M5). From this data, we have obtained population information using age groups (25–29, 30–34, 35–39, …, and > 80) for the 25 administrative districts in Seoul between 2016 and 2020.

#### Fine particulate matter concentration

We used monthly prediction data for air pollution concentrations, estimated at a 1 km^2^ spatial resolution covering all 226 regions nationwide. This data was developed through the AiMS-CREATE (Ai-Machine learning and Statistics Collaborative Research Ensemble for Air pollution, Temperature, and all types of Environmental exposures) network. The AiMS-CREATE network utilized various predictors, including atmospheric, meteorological, and land-use factors. These were obtained from satellite-based data using the Google Earth Engine and Socioeconomic Data and Applications Center. Additionally, it integrated inverse distance-weighted ground-level air pollutant concentrations calculated using national monitoring data alongside regional socioeconomic variables sourced from the Community Health Survey database provided by the Korean Disease Control and Prevention Agency [17]. This prediction model is an ensemble model comprising three machine-learning models (gradient boosting, random forest, and neural networks). A comprehensive description of the construction process for the overall prediction model is available in previous research papers [17–19]. Park et al. reported the performance of a model predicting PM_2.5_, revealing a cross-validated R^2^ of 0.87 [19]. Using this exposure data, we calculated the annual average PM_2.5_ concentration data for Seoul across 25 administrative districts.

### Exposure-response function

For the exposure-response function, we employed the Global Exposure Mortality Model (GEMM) with a nonlinear (i.e., supra-linear) assumption to estimate the health impact assessment attributable to ambient PM_2.5_ exposure [20]. Burnett et al. (2018) suggested a nonlinear exposure-response relationship between ambient PM_2.5_ and all-cause and cause-specific mortality (such as IHD, stroke, chronic obstructive pulmonary disease, acute lower respiratory infection, and lung cancer). This conclusion was drawn from data collected from 41 cohorts spanning 16 countries. The GEMM assigns a relative risk (RR) for each 5-year age group, starting from individuals aged ≥ 25 years (25–29, 30–34, 35–39, …, and > 80) (Fig. S1).

### Health impact assessment

The health impact assessment of air pollution can be divided into health burden and benefits. Health burden encompasses the health impacts caused by existing levels of air pollution, while health benefits refer to the positive outcomes resulting from hypothetical scenarios (e.g., air pollution reduction). The formula for mortality burden owing to ambient air pollution exposure is as follows:

1 $$\:AD=M\:\times\:{PAF}_{burden}\times\:Population$$

$$\:{PAF}_{burden}=\frac{p\left(RR-1\right)}{1+p\left(RR-1\right)}$$

2 $$\:{PAF}_{burden}=1-1/{RR}_{baseline}$$

Here, AD indicates attributable deaths due to ambient PM_2.5_. *M* represents mortality and $\:{PAF}_{burden}$ is population attributable fraction of the health burden. *p* represents prevalence of exposure. We assumed $\:\text{p}=1$ for ambient air pollution, as exposure is considered ubiquitous and the exact prevalence is difficult to quantify. This assumption is consistent with previous studies on the health burden of air pollution and follows the methodology recommended by the WHO AirQ+ tool and prior literature. In our study, $\:{RR}_{baseline}$ represents the RR of IHD mortality based on ambient PM_2.5_ concentrations for each district.

3 $$\:HB=M\:\times\:{PAF}_{benefit}\times\:Population$$

4 $$\:{PAF}_{benefit}=1-\frac{{RR}_{reference}}{{RR}_{baseline}}$$

Here, *HB* indicates health benefits (i.e., avoided deaths) resulting from reductions in ambient PM_2.5_. The $\:{PAF}_{burden}$ is a population attributable fraction of the health benefits. The $\:{RR}_{reference}$ denotes the RR of IHD mortality based on reference concentrations of ambient PM_2.5_ concentrations. In this study, avoidable mortality rates are defined as avoided deaths divided by IHD deaths.

For reference concentrations, we considered the KMOE air quality standards (PM_2.5_: 15 µg/m^3^). Furthermore, mortality benefits were estimated assuming reference concentrations of 10 µg/m^3^ (based on the 2005 WHO AQG) and 5 µg/m^3^ (based on the WHO AQG updated in 2021).

We categorized age groups into ≥ 25, ≥ 45, and ≥ 65 years to calculate the mortality burdens and benefits of IHD attributable to PM_2.5_ exposure across different age groups.

All data pre-processing and analysis were performed using R software version 4.2.1.

## Results

### PM_2.5_ concentration

**Fig. 1:**
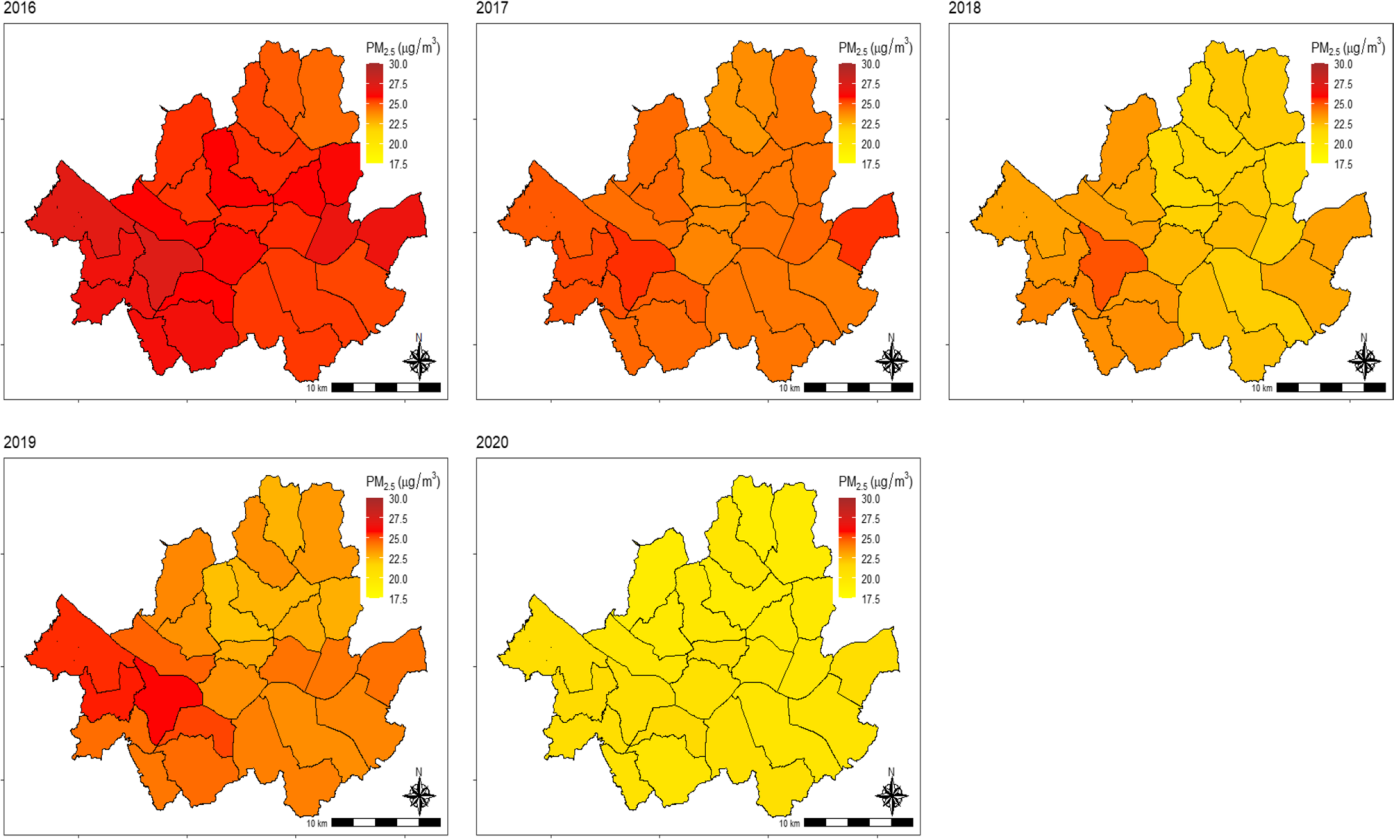
Annual average PM_2.5_ concentration map by districts in Seoul, South Korea from 2016 to 2020. Abbreviations: PM_2.5_, fine particulate matter

The annual average PM_2.5_ concentration in Seoul has steadily decreased each year (Fig. 1 and Table S1). The average PM_2.5_ concentration was 25.9 µg/m^3^ in 2016 and 20.3 µg/m^3^ in 2020, resulting in an overall average of 23.5 µg/m^3^ between 2016 and 2020.

### Mortality pattern in Seoul from 2016 to 2020

Throughout the study, the average population aged ≥ 25, ≥ 45, and ≥ 65 years were approximately 7.4 million, 4.3 million, and 1.3 million, respectively (Table 1). The total number of deaths from IHD was 10,971 (≥ 25 years), 10,671 (≥ 45 years), and 8,341 (≥ 65 years). The mortality rates per 100,000 population were 148.0 (≥ 25 years), 246.3 (≥ 45 years), and 620.1 (≥ 65 years).

**Table 1:** Population, number of IHD deaths, and death rates by year in seoul, South Korea

Year	PM_2.5_ (µg/m^3^)	Population			IHD deaths			Death rates (per 100,000)		
		≥ 25	≥ 45	≥ 65	≥ 25	≥ 45	≥ 65	≥ 25	≥ 45	≥ 65
2016	25.9	7,391,566	4,202,835	1,237,369	2,153	2,075	1,614	29.1	49.4	130.4
2017	24.5	7,392,610	4,273,719	1,283,756	2,107	2,048	1,621	28.5	47.9	126.3
2018	22.7	7,401,551	4,336,531	1,339,178	2,102	2,048	1,589	28.4	47.2	118.7
2019	24.0	7,423,873	4,397,977	1,396,111	2,152	2,091	1,644	29.0	47.5	117.8
2020	20.3	7,461,956	4,454,637	1,468,919	2,457	2,409	1,873	32.9	54.1	127.5
Total	23.5^a^	7,414,311^a^	4,333,140^a^	1,345,067^a^	10,971^b^	10,671^b^	8,341^b^	148.0^b^	246.3^b^	620.1^b^

* Abbreviations:* PM_2.5_, fine particulate matter; *IHD*, Ischemic heart disease

^a^represents the average value for each year; ^b^indicates the sum of deaths or death rates over the study period

### Excess deaths due to PM_2.5_ exposure

Table 2 presents the excess deaths and corresponding rates of IHD attributed to PM_2.5_ exposure in Seoul between 2016 and 2020. For individuals aged ≥ 25 years, an estimated 2,861 excess IHD deaths (95% CI: 2,629–3,087) were attributed to PM_2.5_ exposure. Additionally, among those aged ≥ 45 years, 2,436 excess IHD deaths (95% CI: 2,118–2,741) were attributed to the exposure. For those aged ≥ 65 years, 1,881 excess IHD deaths (95% CI: 1,723–2,034) were estimated to be attributed to the exposure. The excess IHD death rates per 100,000 population were estimated at 38.6 (95% CI: 35.5–41.6) for individuals aged ≥ 25 years, 56.2 (95% CI: 48.99–63.3) for those aged ≥ 45 years, and 139.8 (95% CI: 128.1–151.2) for those aged ≥ 65 years.

**Table 2:** IHD mortality burden due to PM_2.5_ exposure in seoul, South korea, between 2016 and 2020

Year	Excess deaths (95% CI)			Excess death rates^*^ (95% CI)		
	≥ 25	≥ 45	≥ 65	≥ 25	≥ 45	≥ 65
2016	604 (555, 650)	503 (438, 565)	389 (357, 420)	8.2 (7.5, 8.8)	12.0 (10.4, 13.4)	31.4 (28.9, 33.9)
2017	564 (518, 608)	476 (414, 535)	377 (346, 408)	7.6 (7.0, 8.2)	11.1 (9.7, 12.5)	29.4 (27.0, 31.8)
2018	538 (494, 581)	457 (397, 515)	351 (321, 379)	7.3 (6.7, 7.8)	10.5 (9.2, 11.9)	26.2 (24.0, 28.3)
2019	570 (523, 614)	490 (427, 552)	378 (346, 409)	7.7 (7.1, 8.3)	11.2 (9.7, 12.5)	27.1 (24.8, 29.3)
2020	586 (538, 634)	509 (442, 574)	386 (353, 418)	7.9 (7.2, 8.5)	11.4 (9.9, 12.9)	26.3 (24.0, 28.5)
Total^a^	2,861 (2,629, 3,087)	2,436 (2,118, 2,741)	1,881 (1,723, 2,034)	38.6 (35.5, 41.6)	56.2 (48.9, 63.3)	139.8 (128.1, 151.2)

*Abbreviations:* *CI*, confidence interval

^a^indicates total deaths or death rates over the study period

*Death rate per 100,000 population

### Avoided deaths due to PM_2.5_

**Fig. 2:**
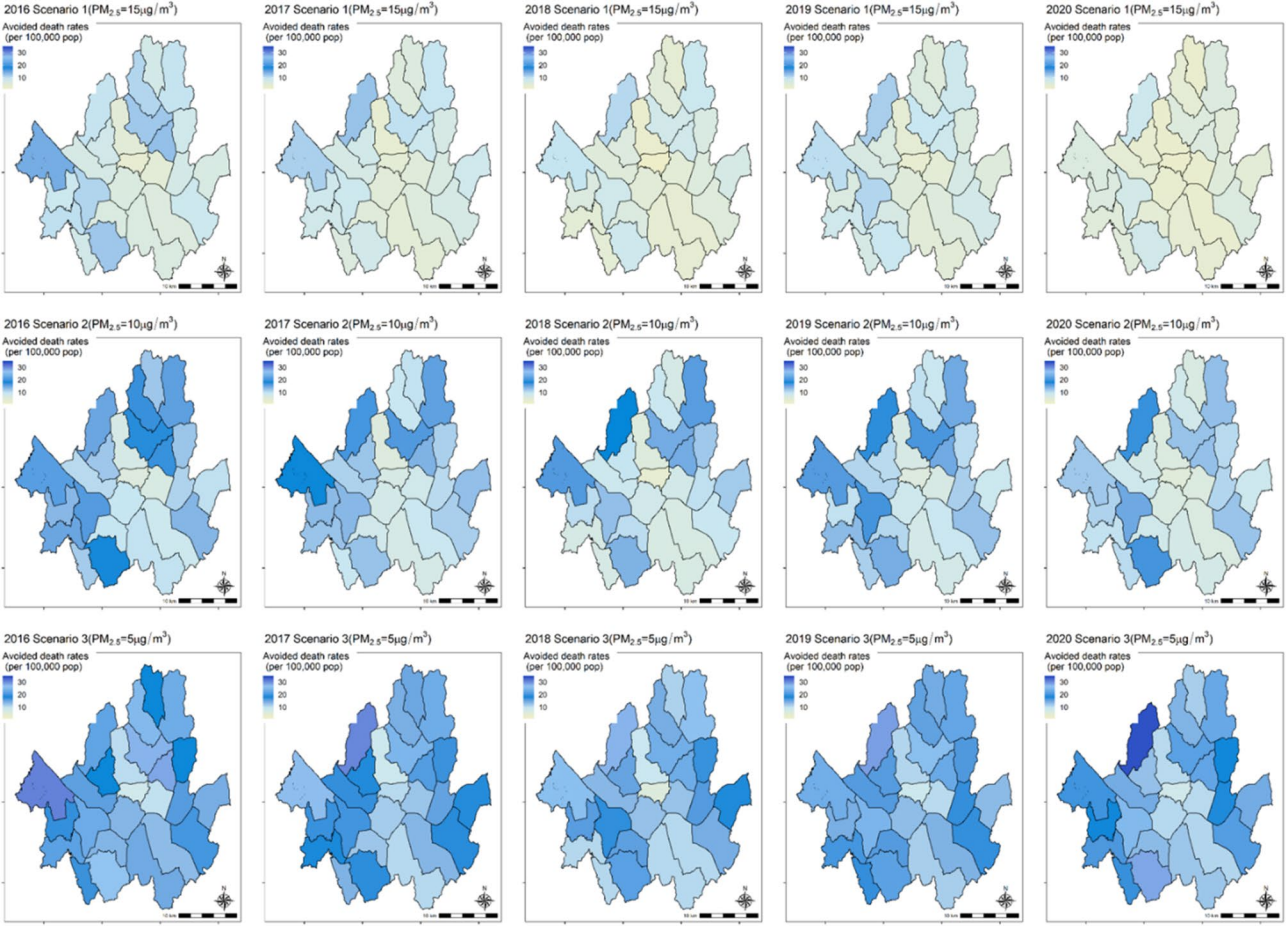
Avoided death rate map due to PM_2.5_ concentration reduction by districts in Seoul, South Korea from 2016–2020

Table 3 shows avoided deaths, avoided rates (per 100,000 population), and avoidable mortality rates. During the study period, achieving a PM_2.5_ concentration of 15 µg/m^3^ was estimated to prevent approximately 837 deaths (95% CI: 761–912) from IHD among individuals aged ≥ 25 years, 740 deaths (95% CI: 639–836) among those aged ≥ 45 years, and 535 deaths (95% CI: 487–585) for those aged ≥ 65 years. The avoided death rates per 100,000 population were higher among the elderly. The avoided deaths rate (per 100,000 population) was estimated at 11.3 (95% CI: 10.3–12.3) for those aged ≥ 25 years, 17.1 (95% CI: 14.7–19.3) for those aged ≥ 45 years, and 39.8 (95% CI: 36.2–43.5) for those aged ≥ 65 years. Assuming a PM_2.5_ concentration of 15 µg/m^3^, the avoidable mortality rates were estimated to be 7.6% (95% CI: 6.9–8.3) for individuals aged ≥ 25 years, 6.9% (95% CI: 6.0–7.8) for those aged ≥ 45 years, and 6.4% (95% CI: 5.8–7.0) for those aged ≥ 65 years. Figure 2 illustrates the avoided death rates (per 100,000 population) for different exposure reduction scenarios (15 µg/m^3^, 10 µg/m^3^, and 5 µg/m^3^). Lower reference concentrations resulted in higher avoided IHD mortality (Table S2–S3).

**Table 3:** IHD mortality benefit for meeting 15 µg/m^3^ in seoul, South korea, between 2016 and 2020

Year	Avoided deaths (95% CI)			Avoided death rates^*^ (95% CI)			Avoidable mortality rates (95% CI)		
	≥ 25	≥ 45	≥ 65	≥ 25	≥ 45	≥ 65	≥ 25	≥ 45	≥ 65
2016	211 (192, 230)	179 (153, 203)	132 (120, 144)	2.9 (2.6, 3.1)	4.3 (3.6, 4.8)	10.7 (9.7, 11.6)	9.8% (8.9, 10.7)	8.6% (7.4, 9.8)	8.2% (7.4, 8.9)
2017	180 (163, 196)	156 (133,177)	117 (107, 128)	2.4 (2.2, 2.6)	3.7 (3.1, 4.1)	9.1 (8.3, 10.0)	8.5% (7.8, 9.3)	7.6% (6.5, 8.6)	7.2% (6.6, 7.9)
2018	149 (135, 162)	133 (144, 151)	95 (86, 103)	2.0 (1.8, 2.2)	3.1 (2.6, 3.5)	7.1 (6.4, 7.7)	7.1% (6.4, 7.7)	6.5% (5.6, 7.4)	6.0% (5.4, 6.5)
2019	175 (159, 191)	164 (141, 187)	113 (103, 124)	2.4 (2.1, 2.6)	3.7 (3.2, 4.3)	8.1 (7.4, 8.9)	8.1% (7.4, 8.9)	7.8% (6.7, 8.9)	6.9% (6.3, 7.5)
2020	122 (111, 134)	108 (98, 118)	78 (71, 86)	1.6 (1.5, 1.8)	2.4 (2.2, 2.6)	5.3 (4.8, 5.9)	5.0% (4.5, 5.4)	4.5% (4.1, 4.9)	4.2% (3.8, 4.6)
Total^a^	837 (761, 912)	740 (639, 836)	535 (487, 585)	11.3 (10.3, 12.3)	17.1 (14.7, 19.3)	39.8 (36.2, 43.5)	7.6% (6.9, 8.3)	6.9% (6.0, 7.8)	6.4% (5.8, 7*.*0)

*Abbreviations:* *CI*, confidence interval

^a^indicates total deaths or death rates over the study period

*Death rate per 100,000 population

## Discussion

### Main results

We estimated the IHD mortality burden attributable to ambient PM_2.5_ and the IHD mortality benefits associated with reductions in PM_2.5_ concentration in Seoul, South Korea, between 2016 and 2020. Our analysis revealed that over 5 years, 2,861 IHD deaths (≥ 25) were associated with PM_2.5_ exposure.

Under the PM_2.5_ reduction scenario, achieving a PM_2.5_ concentration of 15 µg/m^3^ corresponds to an avoidable mortality rate of approximately 8%. Our findings revealed that greater reductions in PM_2.5_ lead to higher avoidable mortality rates.

### Aging population

In a study using South Korean health insurance data between 2002 and 2018, Kim reported an annual increase in hospitalizations due to IHD despite a decreasing trend in the age-standardized hospitalization rate. This suggests that as the population ages, the health burden of IHD among the elderly increases. The estimated health benefits of reducing air pollution vary based on the exposed population, mortality rates, and exposure-response relationship between PM_2.5_ and health outcomes. With population aging, the health burden among the elderly increases owing to the elevated risk of IHD mortality.

### Particulate matter management in Seoul

In December, 2019, the Seoul Metropolitan Government (SMG) announced the implementation of the Seasonal Particulate Matter (PM) Management [21]. This initiative is designed to strictly reduce and manage air pollutant emissions from December to March, a period characterized by high PM concentrations due to seasonal factors. To ensure its effectiveness, the SMG implemented nine tasks and seven supporting actions. Seasonal PM management was first launched between December 2019 and March 2020 and has been conducted annually since then. Following this initiative, on December 1, 2023, the fifth Seasonal PM Management plan (between December 1, 2023 and March 31, 2024) was announced, comprising 16 measures across four areas: reducing exposure from transport, heating, and business sites, as well as minimizing population exposure. According to the 2024 announcement by the SMG, the PM_2.5_ concentration in Seoul has reached its lowest level since the introduction of the seasonal management system in 2019 [22]. During the Seasonal PM management period, a marked improvement in air quality is observed. The annual average PM_2.5_ concentrations dropped from 35 µg/m^3^ during the pre-implementation period (December 2018 to March 2019) to 22 µg/m^3^ in the fifth management period (December 2023 to March 2024). Therefore, we predict that these measures to reduce PM_2.5_ will lower future IHD mortality and increase health benefits.

### Strength

This study has several strengths. First, we employed high-resolution PM_2.5_ concentration data constructed using machine learning techniques, providing a more sophisticated assessment of exposure. Second, we estimated health benefits based on three reference concentrations: (1) KMOE (PM_2.5_: 15 µg/m^3^), (2) 2005 WHO AQG (PM_2.5_: 10 µg/m^3^), and (3) 2021 WHO AQG (PM_2.5_: 5 µg/m^3^). Our findings suggest that as PM_2.5_ concentrations decrease, the rates of avoidable mortality increase.

### Limitations

There are some limitations in this study. First, misclassification issues related to exposure assignment may exist. While we linked exposure at the district level, this may not accurately reflect actual exposure. Therefore, future studies may require more precise spatial resolution for exposure allocation. Second, the exposure-response functions we used are widely employed in global research and, combine data from 41 cohort studies. However, most of these studies primarily focused on regions with lower exposure levels, such as Europe, the United States, and Canada. Therefore, the GEMM exposure-response function may not fully capture the PM_2.5_ and mortality exposure-response relationship in the Asian region. Therefore, future research should employ exposure-response functions aggregated from studies conducted in Asian regions, including South Korea, to evaluate the health impacts of long-term exposure to PM_2.5_ on mortality. Finally, PM_2.5_ consists of various chemical constituents originating from multiple sources, and the associated health effects may differ depending on its composition [23]. However, our study did not directly assess individual PM_2.5_ constituents and estimate their component-specific disease burden, which is limitation. Future studies should aim to analyses the major constituents of PM_2.5_ and estimate their specific health impacts and related disease burden, which would provide more refined evidence for targeted policy interventions.

## Conclusions

This study assessed the burden of IHD mortality attributable to ambient PM_2.5_ and the associated health benefits of reducing PM_2_._5_ concentrations in Seoul, South Korea, from 2016 to 2020. Our findings indicate that reducing ambient PM_2.5_ concentrations could lead to substantial reductions in IHD-related mortality. Given the projected growth of the aging population, proactive air quality interventions may yield increasingly important public health benefits in the future.

## Supplementary Information

Supplementary Material 1.

## Data Availability

No datasets were generated or analysed during the current study.

## Abbreviations

AQG: Air quality guideline
AiMS-CREATE: Ai-Machine learning and Statistics Collaborative Research Ensemble for Air pollution, Temperature, and all types of Environmental exposures
CVD: cardiovascular disease
DALYs: disability-adjusted life years
PM2.5: particulate matter
GEMM: Global Exposure Mortality Model
IHD: ischemic heart disease
KMOE: Korean Ministry of Environment
PM: particulate matter (PM)
RR: relative risk
SMG: Seoul Metropolitan Government
WHO: World Health Organization
