# Supplementary material for: Ischemic heart disease mortality due to fine particulate matter in Seoul between 2016 and 2020
Source: BMC Public Health. 2025 Oct 22;25:3575. doi: 10.1186/s12889-025-24204-y (PMC12542303; doi:10.1186/s12889-025-24204-y)
Supplement: Supplementary file 1 — Supplementary Material 1. [file 12889_2025_24204_MOESM1_ESM.docx]

**Appendix**

**Table S1.** Annual average of PM_2.5_ concentrations by districts in Seoul, South Korea, between 2016 and 2020.

| Districts of Seoul | PM_2.5_ (μg/m^3^) | | | | |
| --- | --- | --- | --- | --- | --- |
|  | 2016 | 2017 | 2018 | 2019 | 2020 |
| Jongno-gu | 25.9 | 23.7 | 21.3 | 22.3 | 20.0 |
| Jung-gu | 25.6 | 23.8 | 21.9 | 23.0 | 21.0 |
| Yongsan-gu | 26.1 | 23.9 | 22.6 | 23.7 | 20.7 |
| Seongdong-gu | 25.6 | 24.4 | 22.5 | 24.4 | 20.3 |
| Gwangjin-gu | 26.7 | 24.6 | 21.9 | 24.3 | 20.1 |
| Dongdaemun-gu | 25.9 | 24.2 | 22.1 | 23.0 | 19.8 |
| Jungnang-gu | 26.1 | 24.4 | 21.4 | 22.8 | 19.8 |
| Seongbuk-gu | 25.5 | 24.3 | 21.8 | 22.7 | 19.6 |
| Gangbuk-gu | 25.3 | 23.5 | 21.6 | 23.7 | 20.2 |
| Dobong-gu | 24.9 | 23.7 | 22.1 | 22.7 | 19.4 |
| Nowon-gu | 24.6 | 24.3 | 22.0 | 23.4 | 19.6 |
| Eunpyeong-gu | 25.5 | 24.5 | 23.4 | 23.9 | 19.5 |
| Seodaemun-gu | 25.5 | 24.6 | 23.0 | 23.6 | 19.8 |
| Mapo-gu | 25.9 | 24.5 | 23.3 | 24.7 | 20.2 |
| Yangcheon-gu | 26.5 | 25.3 | 23.5 | 25.7 | 21.2 |
| Gangseo-gu | 27.1 | 24.9 | 23.2 | 25.6 | 20.9 |
| Guro-gu | 26.6 | 25.1 | 23.9 | 24.5 | 21.0 |
| Gumcheon-gu | 26.3 | 24.6 | 23.7 | 24.3 | 20.6 |
| Yeongdeungpo-gu | 27.3 | 25.6 | 25.0 | 25.9 | 20.9 |
| Dongjak-gu | 25.8 | 24.9 | 23.4 | 25.3 | 21.3 |
| Gwanak-gu | 26.4 | 24.6 | 23.7 | 24.5 | 20.2 |
| Seocho-gu | 25.4 | 24.3 | 22.4 | 24.1 | 20.7 |
| Gangnam-gu | 25.4 | 24.3 | 21.9 | 23.7 | 20.2 |
| Songpa-gu | 25.2 | 24.1 | 23.0 | 23.9 | 20.3 |
| Gangdong-gu | 26.6 | 25.5 | 23.2 | 24.4 | 21.0 |
| Total | 25.9 | 24.5 | 22.7 | 24.0 | 20.3 |

**Abbreviations**: PM_2.5_, fine particulate matter;

**Table S2.** IHD mortality benefit for meeting 10 μg/m^3^ (2005 WHO AQG) in Seoul, South Korea between 2016 and 2020.

| Year | Avoided deaths (95% CI) | | | Avoided death rates^*^ (95% CI) | | | Avoidable mortality rates (95% CI) | | |
| --- | --- | --- | --- | --- | --- | --- | --- | --- | --- |
|  | ≥25 | ≥45 | ≥65 | ≥25 | ≥45 | ≥65 | ≥25 | ≥45 | ≥65 |
| 2016 | 322  (294, 350) | 300  (274, 327) | 203  (185, 221) | 4.4  (4.0, 4.7) | 7.1  (6.5, 7.8) | 16.4  (15.0, 17.9) | 15.0% (13.7, 16.2) | 14.5% (13.2, 15.7) | 9.8%  (8.9, 10.6) |
| 2017 | 288  (262, 313) | 273  (249, 296) | 189  (172, 206) | 3.9  (3.5, 4.2) | 6.4  (5.8, 6.9) | 14.7  (13.4, 16.0) | 13.7% (12.4, 14.9) | 13.3% (12.1, 14.5) | 9.2%  (8.4, 10.0) |
| 2018 | 257  (236, 311) | 245  (225, 267) | 165  (150, 180) | 3.5  (3.2, 3.8) | 5.7  (5.2, 6.2) | 12.3  (11.2, 13.4) | 12.2% (11.2, 13.4) | 12.0% (11.0, 13.1) | 8.1%  (7.3, 8.8) |
| 2019 | 287  (261, 311) | 272  (248, 294) | 187  (170, 203) | 3.9  (3.5, 4.2) | 6.2  (5.6, 6.7) | 13.4  (12.2, 14.5) | 13.3% (12.1, 14.4) | 13.0% (11.8, 14.1) | 8.9%  (8.1, 9.7) |
| 2020 | 252  (231, 275) | 243  (222, 265) | 162  (149, 178) | 3.4  (3.1, 3.7) | 5.4  (5.0, 5.9) | 11.1  (10.1, 12.1) | 10.3%  (9.4, 11.2) | 10.1%  (9.2, 11.0) | 6.7%  (6.2, 7.4) |
| Total^a^ | 1,407 (1282, 1529) | 1,333 (1217, 1450) | 906  (826, 987) | 19.0  (17.3, 20.6) | 30.8  (28.1, 33.5) | 67.4  (61.4, 73.4) | 12.8% (11.7, 13.9) | 12.5% (11.4, 13.6) | 8.5%  (7.7, 9.2) |

**Abbreviations**: CI, confidence interval

*Death rate per 100,000 population

**Table S3.** IHD mortality benefit for meeting 5 μg/m^3^ (2021 WHO AQG) in Seoul, South Korea between 2016 and 2020.

| Year | Avoided deaths (95% CI) | | | Avoided death rates^*^ (95% CI) | | | Avoidable mortality rates (95% CI) | | |
| --- | --- | --- | --- | --- | --- | --- | --- | --- | --- |
|  | ≥25 | ≥45 | ≥65 | ≥25 | ≥45 | ≥65 | ≥25 | ≥45 | ≥65 |
| 2016 | 469  (430, 508) | 439  (402, 476) | 299  (273, 324) | 6.3  (5.8, 6.9) | 10.4  (9.6, 11.3) | 24.2  (22.1, 26.2) | 21.8%  (19.9, 23.6) | 21.1% (19.3, 22.9) | 14.4% (13.2, 15.6) |
| 2017 | 431  (395, 468) | 409  (375, 445) | 286  (262, 311) | 5.8  (5.3, 6.3) | 9.6  (8.8, 10.4) | 22.3  (20.4, 24.2) | 20.5%  (18.8, 22.2) | 20.0% (18.3, 21.7) | 14.0% (12.8, 15.2) |
| 2018 | 404  (371, 438) | 385  (353, 518) | 261  (239, 284) | 5.5  (5.0, 5.9) | 8.9  (8.1, 9.6) | 19.5  (17.8, 21.2) | 19.2%  (17.6, 20.8) | 18.8% (17.3, 20.4) | 12.7% (11.7, 13.9) |
| 2019 | 434  (396, 470) | 412  (377, 446) | 285  (261, 310) | 5.8  (5.3, 6.3) | 9.4  (8.6, 10.1) | 20.4  (18.7, 22.2) | 20.2%  (18.4, 21.8) | 19.7% (18.0, 21.3) | 13.6% (12.5, 14.8) |
| 2020 | 427  (390, 463) | 412  (376, 447) | 278  (254, 302) | 5.7  (5.2, 6.2) | 9.3  (8.4, 10.0) | 18.9  (17.3, 20.6) | 17.4%  (15.9, 18.9) | 17.1% (15.6, 18.6) | 11.6% (10.5, 12.5) |
| Total^a^ | 2,166  (1981, 2347) | 2,057 (1882, 2231) | 1,410 (1288, 1532) | 29.2  (26.7, 31.7) | 47.5  (43.4, 51.5) | 104.8 (95.8, 113.9) | 19.7%  (18.1, 21.4) | 19.3% (17.6, 20.9) | 13.2% (12.1, 14.4) |

**Abbreviations**: CI, confidence interval

*Death rate per 100,000 population


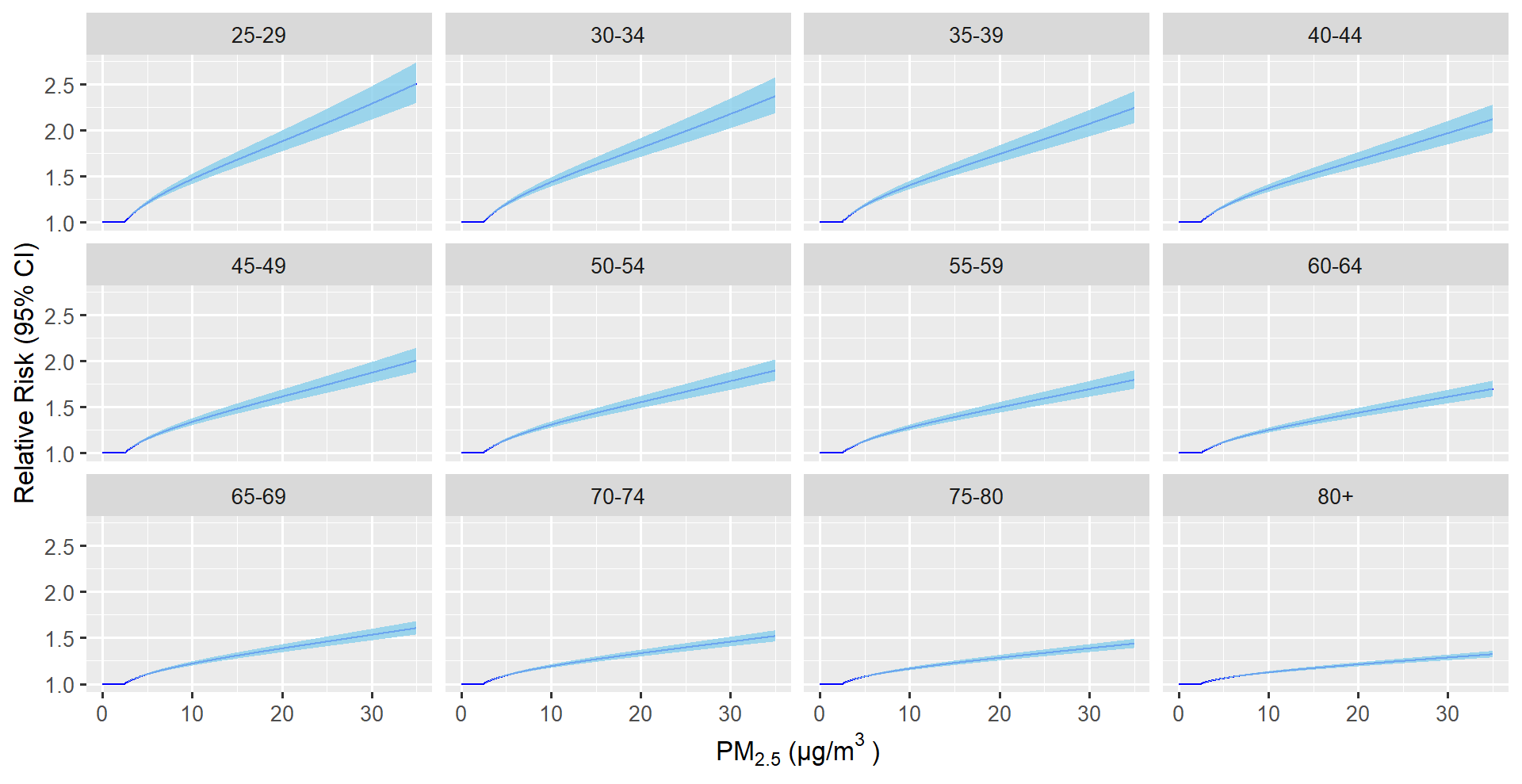


**Figure S1.** Exposure-response association between PM_2.5_ exposure and IHD mortality based on age group using the GEMM.
